# From Chemist's Shop to Physic's Throne

**Published:** 1893-09-16

**Authors:** 


### From Chemist's Shop to Physic's Throne.

A middle-aged practitioner of medicine, a former student of University College Hospital, was speaking to the writer the other day in terms of enthusiasm of Sir William Jenner, and among other things he said: "Sir William in his lectures often used to encourage the students to strenuousness in their work by telling them that he himself had commenced his career as an apprentice in a chemist's shop." Sir William Jenner's career as a practising physician is now closed, but he is enjoying a period of *otium cum dignitate* as an English country gentleman, which we hope may last for many years. The boy of the chemist's shop, before he retired from the active practice of his profession, had been seven years—namely, from 1881 to 1887—President of the Royal College of Physicians of London.

It is a maxim well worth remembering that "What one man has done another man may do." This is not the same as saying that every chemist's apprentice who forsakes the compounding and the sale of drugs for the dissecting-room and the physiological laboratory will become President of the College of Physicians; far from it. But it is a note of promise that if a young man will but do his best he is certain either to achieve success or to deserve it; and next to achieving success there is nothing in the world so entirely satisfactory as deserving it.

Sir William Jenner was a great prescriber in his day, and no doubt his prescriptions occasionally failed, like other people's. If he were asked to give a prescription for turning chemists' apprentices into medical baronets, he would probably decline. But though he would decline to prescribe he might verv properly say, "I have given you an object lesson; make the best use you can of that." And this is so. Whether we think much or little of medical baronets, in general, and of Sir William Jenner in particular, we cannot deny that it is a very considerable performance and means a good deal of capacity of some kind when a tradesman's apprentice achieves the dignity of a medical baronetcy with universal approval.

Is it a good thing for a medical student to set his mind on achieving a baronetcy, or the Presidency of the Royal College of Physicians? This is coming to business. Most people would say at once that of course it is a good thing, for if a young man does but aim high enough he is sure to reach at least a respectable elevation. The writer, however, is inclined to think that on the whole it is not a good thing for so young a man as the average medical student to set his mind upon a particular post of honour and to resolve that that and nothing else shall be the goal of his life. It is very likely that when Sir William Jenner was a medical student he never dreamt of the presidency of the College of Physicians, or of a baronetcy; if he had he would certainly have given himself many unhappy hours, and not improbably the very determination to reach those particular distinctions would have made him lose them.

The idea of the present writer is that the period of student life ought to be a very happy—or if the reader prefers it—a very "jolly" one. It cannot be "jolly" if the soul be eaten up by ambition. The student period ought to be complete in itself. "I am going to be a medical student for five years," the young man may say; "I will make the most I can of that;" and the way to make the most of it is to do its work and to enjoy its pleasures. Nevertheless youth will look forward; it will fix its mind upon conspicuous examples of persons who have pursued its own lines in life. Sir William Jenner is an object lesson well worth looking at, and the consideration of him and his career can do young medical students nothing but good.

Sir William Jenner had one powerful and faithful friend in life, and it was mainly through that friend that he was started on his career and achieved such distinction in it. Who was this friend and patron? It was himself! Neither great connections nor abundance of money had Jenner in his youth or manhood. But he had something which was better, more trustworthy, more certain to succeed than either: he had determination, industry, and sense. "Work," say the mentors of youth; "work and evermore work." "Work in your labour and work in your leisure," said one thirty years ago who has since made himself a considerable place in the world. Well, that is all right up to a certain point. But the counsel which points to eternal work is something of the nature of a brickbat; it is of hard consistency, and when it is flung at the head of a man who has already worked too much and too long, as some medical students have done, it is apt to hit him hard and to hurt him a good deal. Work by all means, and work strenuously, conqueringly, magnificently when you are at it. But play well also; play, young men, by all means; and when you are playing let the work, and the examinations—and the examiners, too—all "go to Hongkong," so far as you are concerned.

The man who has no counsel to offer to young men but that of eternal work is usually somewhat lean in the soul. The truth is, we cannot work always, and if we did we should be exceedingly dull and provokingly uninteresting. We must think a little as well as work. One reason why a great many very deserving medical students fail of their ambition is because they work too much and think too little. In medical practice the case is still worse. A great many very hard workers prove complete failures for this very reason. They have done an infinity of work, they possess an infinity of knowledge; but because they have never accustomed themselves to independent thinking, they do not know how to bring their knowledge to the most profitable market. They are like savages in Central Africa, who would be poor with all the wealth of the Bank of England if they had no natural resources of their own. Every medical student, and every practitioner, too, should lay this thoroughly to heart, that the merely "crammed" man is very seldom indeed the successful man. He is too much like the crammed chicken, stuffed and helpless, and only fit to be roasted.

Sir William Jenner has certainly been a hard worker, but he has been a hard thinker, too; and, above all, he has been a man of sense and conduct. Without sense and conduct the most brilliant man in the world is simply nowhere before the long life-race draws to its close. In the affairs of the body, the thing that stands wear and tear is the constitution; and in the affairs of the mind and of achievement in life it is the conduct. What a man does, and does over a term of years, is what raises him to honour or thrusts him down to oblivion, if not to disgrace. There is a fact which is worthy of a great deal of consideration on the part of young students, who have no experience to guide them. It is this, that whilst every middle-aged person has known numbers of brilliant youths who have entirely failed in after-life, nobody ever yet saw a person whose strength lay in his conduct make a failure of himself. This is a law in all human affairs, and a law experimentally established. To aim at being a great doctor without also aiming at being a great man, is one of the most hopeless and impossible tasks a man can set himself upon. The doctor never makes the man, but the man often—perhaps always—makes the doctor. Character is the soul of intellect, as it is also the basis and energy of achievement. Medicine, like every other profession and calling, can furnish many examples of men who have risen to the highest honour by industry, sense, and conduct. There are few such men who better deserve the study and imitation of medical students than Sir William Jenner.

